# Prescriptions

**Published:** 1893-06

**Authors:** 


					﻿. Prescription Department.
OVARIAN NEURALGIA.
B. Tinct digitalis, 3 j.
Tinct gelsemii, 3 ss.
Potassii bromidi § ss.
Aquae, 5 vj. M.
Sig. Tablespoonful in water every
three hours.
DYSENTERY.
B. Catchechu pulv. 3 ij.
Pulv. acacia, § ss.
Aquae, §vj.
M. Sig. A teaspoonful every two
hours.—Niemeyer.
GONORRHOEA.
B. Soda Biborate, 3 iss.
Morphia Sulph., gr. v.
Listerine, § iss.
Aq. Rosae, q. s., J vi.
Mix. Sig. Use an injection three
times a day.
. NERVOUS DEBILITY.
B. Acid. Phos., Dilut, 3 iv.
Calisayoe Elix., § ij-
Elix. Valerian. Ammon., J j.
Glycerini, 5 ij-
Vini xerici, q. s., 5 viij.
Mix. Sig. Tablespoonful in water
three times a day.
DYSPEPSIA.
B—Subnitrate of Bismuth, 150 grs.
Sulphate of Magnesia, 150 grs.
Prepared Chalk...........150 grs.
Phosphate of Lime. . . .150 grs.
Sig. Divide into 40 powders. One
before each meal.
Inhalation for Asthma.—During
the attack the following :
B—Ether..............1 ounce.
01. Terebinth....4 drachms.
Acidi Benzoici.... 4 drachms.
Balsam, Tolutani. .2 drachms
M. Sig. For inhalation.
CHRONIC DIARRHCEA WITH INTESTINAL,
FERMENTATION.
B. Salol................ 3	parts.
Castor Oil.......... 15	parts.
Rhubarb.............30	parts.
Gum Arabic.......... 10	parts.
Water.......’.....120 parts.
Mix. A tablespoonful every hour
till the bowels move.—Lo Prog. Mod.
FOR PAIN EROM FLATULENCE OR COLIC.
By Dr. N. R. Adams, Collingswood,
N. J.
B. Morphia sulphate, gr j.
Chloroform, 3 ss.
Soda bicarbonate, 3 ij
Syrup zingberis, § ss.
Aqua menth. pip., 5 Jss*
Sig. Teaspoonful in hot water every
twenty to thirty minutes until relieved.
A GOOD COUGH MIXTURE.
B. Ammonia. Muriat., 3 iiss.
Spts. Chloroform, 3 vi.
Tr. Opii Camph.,
Tr. Hyoscyami, aa 5 ]•
Syr. Tolu, q. s. ad, 5 iy,
M. Sig. Two teaspoonfuls in water
every 2, 3 or 4 hours.
EXPECTORANT FOR ACUTE BRONCHITIS.
B. Ol. terebin th. 3 ij—iij.
Muc. acacias, q. s.
Aq. cinnamon, 5 ]•
Aquae, q. s. ad, ^yvj. M.
Sig. Tablespoonful in water every
four hours.
INTESTINAL ANTISEPSIS.
B. Antikamnia and Salol Tablets aa
grs, v. Num. 24. Sig. One
every three or four hours.
				

